# Clinical and biochemical footprints of inherited metabolic disease. XVI. Hematological abnormalities

**DOI:** 10.1016/j.ymgme.2023.107735

**Published:** 2023-11-13

**Authors:** Eoghan Dunlea, Ellen Crushell, Melanie Cotter, Nenad Blau, Carlos R. Ferreira

**Affiliations:** a Dept of Haematology, Children’s Health Ireland, Temple Street, Dublin, Ireland; b School of Medicine, Trinity College, Dublin, Ireland; c National Centre for Inherited Metabolic Disorders, Children’s Health Ireland, Temple Street, Dublin, Ireland; d School of Medicine, University College Dublin, Dublin, Ireland; e Division of Metabolism, University Children’s Hospital, Zürich, Switzerland; f National Human Genome Research Institute, National Institutes of Health, Bethesda, MD, USA

**Keywords:** Bone marrow morphology, White blood cell morphology, Neutropenia, Thrombocytopenia, Pancytopenia, Anemia, Coagulation abnormalities, IEMbase

## Abstract

Many classical inherited metabolic diseases (IMDs) are associated with significant hematological complications such as anemia or thrombosis. While these may not be the prominent presenting feature of these conditions, management of these issues is important for optimal outcomes in people with IMDs. Some disorders that are included in the nosology of inherited metabolic disorders, such as inherited disorders of red cell energy metabolism, have purely hematological features, and have typically been cared for by a hematologist. In the 16th issue of the Footprints series, we identified 265 IMDs associated with hematological abnormalities. We review the major hematological manifestations of IMDs, suggest further investigation of hematological findings, and discuss treatment options available for specific hematological complications of IMDs.

## 1. Introduction

This is the 16th in a series of articles that provide a comprehensive list of inherited metabolic disorders (IMDs), identifying associated signs and symptoms according to system involvement. Previous articles have dealt with movement disorders, metabolic liver diseases, psychiatric presentation, metabolic cardiovascular diseases, cerebral palsy phenotype, metabolic dermatoses, ocular phenotype, neoplasia, metabolic ear disorders, metabolic myopathies, gastrointestinal symptoms, immunological defects, respiratory manifestations and kidney disorders associated with IMDs [1–14]. In this issue, we focus on hematological abnormalities, which are frequently found in patients with inherited metabolic disease.

We reviewed the list of inherited metabolic disorders looking for associations with hematological features and identified 265 IMDs associated with hematological abnormalities. Many classical IMDs are associated with significant hematological complications such as anemia or thrombosis. While these may not be the prominent presenting feature of these conditions, management of these issues is important for optimal outcomes in people with IMDs. In certain IMDs, the hematological findings can be helpful in reaching a diagnosis; for example, the presence of vacuolated lymphocytes where a lysosomal storage disorder is suspected. Some IMDs can initially present to a hematologist, for example, neutropenia associated with Barth syndrome, or megaloblastic anemia in infancy due to transcobalamin II deficiency. For this reason, it is important that hematologists include IMDs in the differential diagnosis. Some disorders that are included in the nosology of IMDs, such as inherited disorders of red cell energy metabolism, have purely hematological features, and have typically been cared for by a hematologist. We review the major hematological manifestations of IMDs, suggest further investigation of hematological findings in relation to IMDs, and discuss treatment options available for specific hematological complications of IMDs.

## 2. Materials and methods

The source of the information was IEMbase, a knowledgebase of IMDs (http://www.iembase.org) [15]. As of August 14, 2023, IEMbase tabulates 1880 IMDs and 4107 corresponding clinical and biochemical signs and symptoms which have been grouped into 22 organ systems and conditions (Autonomic system, Cardiovascular, Dental, Dermatological, Digestive, Dysmorphic, Ear, Endocrine, Eye, Genitourinary, Hair, Hematological, Immunological, Metabolic, Muscular, Neurologic, Psychiatric, Kidney, Respiratory, Skeletal, Tumoral and Other). The clinical symptoms associated with hematological abnormalities (*n* = 64) were extracted from the ‘Hematological’ group. The nosology of IMDs [16] was reclassified according to the International Classification of Inherited Metabolic Disorders, ICIMD [17].

### 2.1. Signs and symptoms

We categorized the hematological features of IMDs into 7 different categories, namely, a) abnormal blood cell morphology, b) coagulation abnormalities including bleeding tendency, c) anemias, d) neutropenia, lymphopenia, pancytopenia and thrombocytopenia, e) hypercoagulability (or thrombotic tendency), f) bone marrow abnormality and g) other (Supplemental Table S1). We will highlight the key IMDs which have these features and discuss them in the context of the most prominent or frequent hematological abnormality.

**Fig. 1.**
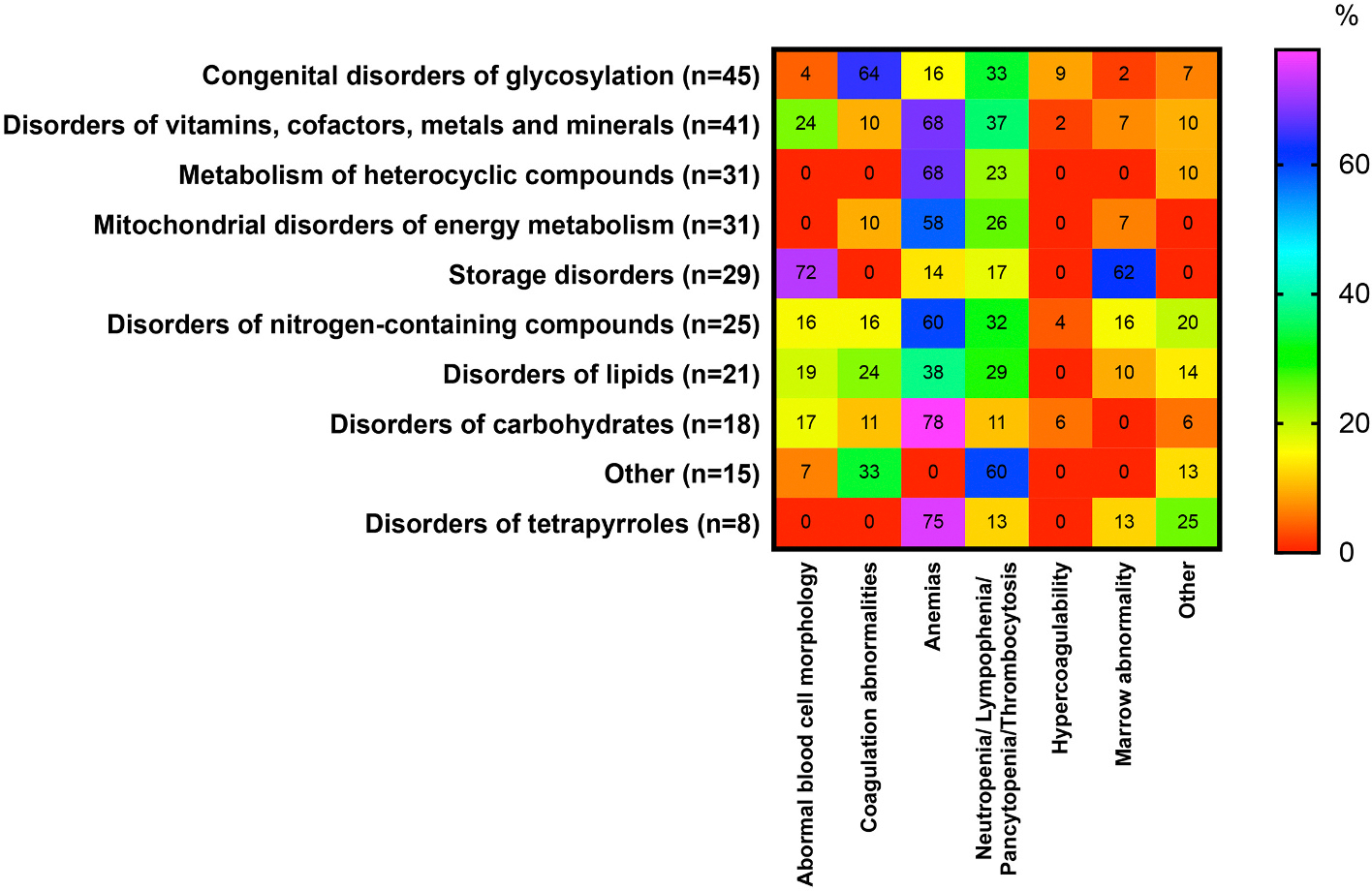
Occurrence (%) of symptoms associated with disorders presenting with hematological phenotype in 10 categories of IMDs. The percentages for hematological abnormalities were calculated using as the denominator the total number of IMDs in each category presenting with any with hematological phenotype. Heat scale ranges from red (0%) for diseases with no particular symptoms reported to violet (100%) for diseases with particular symptoms occurring with highly frequency within the disorders group. For definition of 10 categories of disorders affecting eye see Supplemental Table 2. For interpretation of the references to colour in this figure legend, the reader is referred to the web version of this article. (For interpretation of the references to colour in this figure legend, the reader is referred to the web version of this article.)

**Fig. 2.**
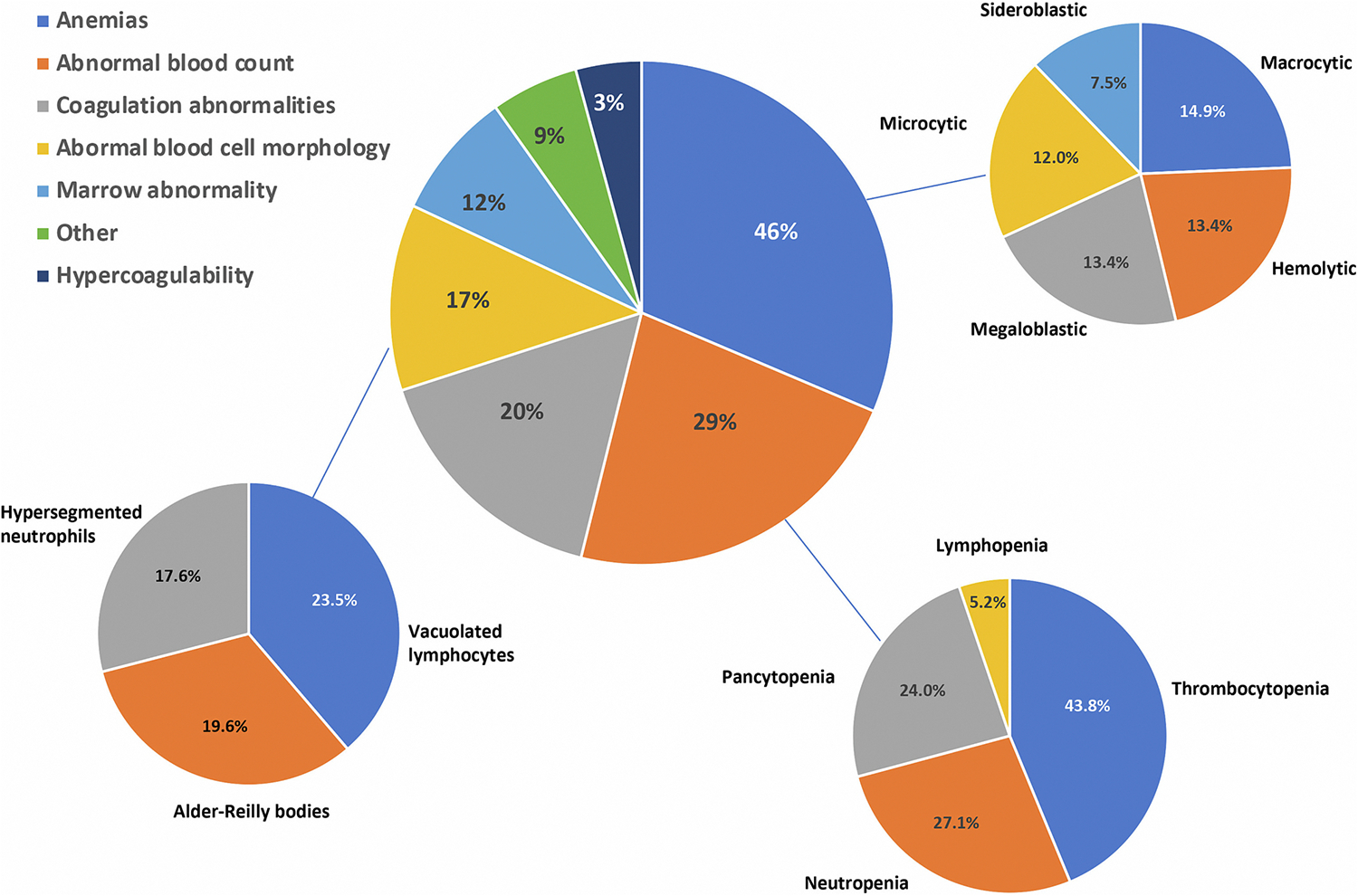
Frequency of hematological abnormalities among 7 signs and symptoms groups and most common hematological abnormalities within Anemias, Abnormal blood count (neutropenia, lymphopenia, pancytopenia, thrombocytosis) and Abnormal blood cell morphology. For details see Supplemental Tables S1 and S2.

Regarding the clinical signs, anemias were the most common hematological abnormality reported in 121/265 (46%) disorders, followed by abnormal blood count 76/265 (29%), coagulation abnormalities 52/265 (20%), marrow abnormalities 31/265 (12%) and hypercoagulability or thrombotic tendency 7/265 (3%) (Fig. 1 and Supplemental Table S2). While anemias may be caused by many different disorders, some hematological findings are more specific to particular disorders e. g., sideroblastic anemia pointing towards mitochondrial disease. A more detailed distribution of hematological signs and symptoms among 7 groups is shown in Fig. 2.

### 2.2. Investigations

Classical hematological and biochemical investigations (Table 1) continue to have a major role in the diagnosis of metabolic and hematological disorders; however, the expansion of next generation genetic testing techniques has hugely helped in the diagnosis of these disorders in the clinic. As these techniques become more available and accessible, the knowledge base will continue to grow in coming years.

### 2.3. Abnormal blood cell/bone marrow morphology

Abnormal cell morphology on the blood film or bone marrow biopsy can be the first indicator of an IMD. A blood film is a rapid, inexpensive, and non-invasive investigation which should always be considered as part of a workup for a suspected metabolic disorder. Findings such as vacuolated lymphocytes can suggest a diagnosis of a lysosomal storage disorder. However, it should be noted that the absence of findings does not exclude a metabolic disorder. Blood films are subject to a significant degree of inter-observer variability and should ideally be examined by an experienced hematologist. It is critically important to provide clinical details with the request, as subtle abnormalities are easily missed unless specifically looked for.

#### 2.3.1. White blood cell morphology

**Fig. 3.**
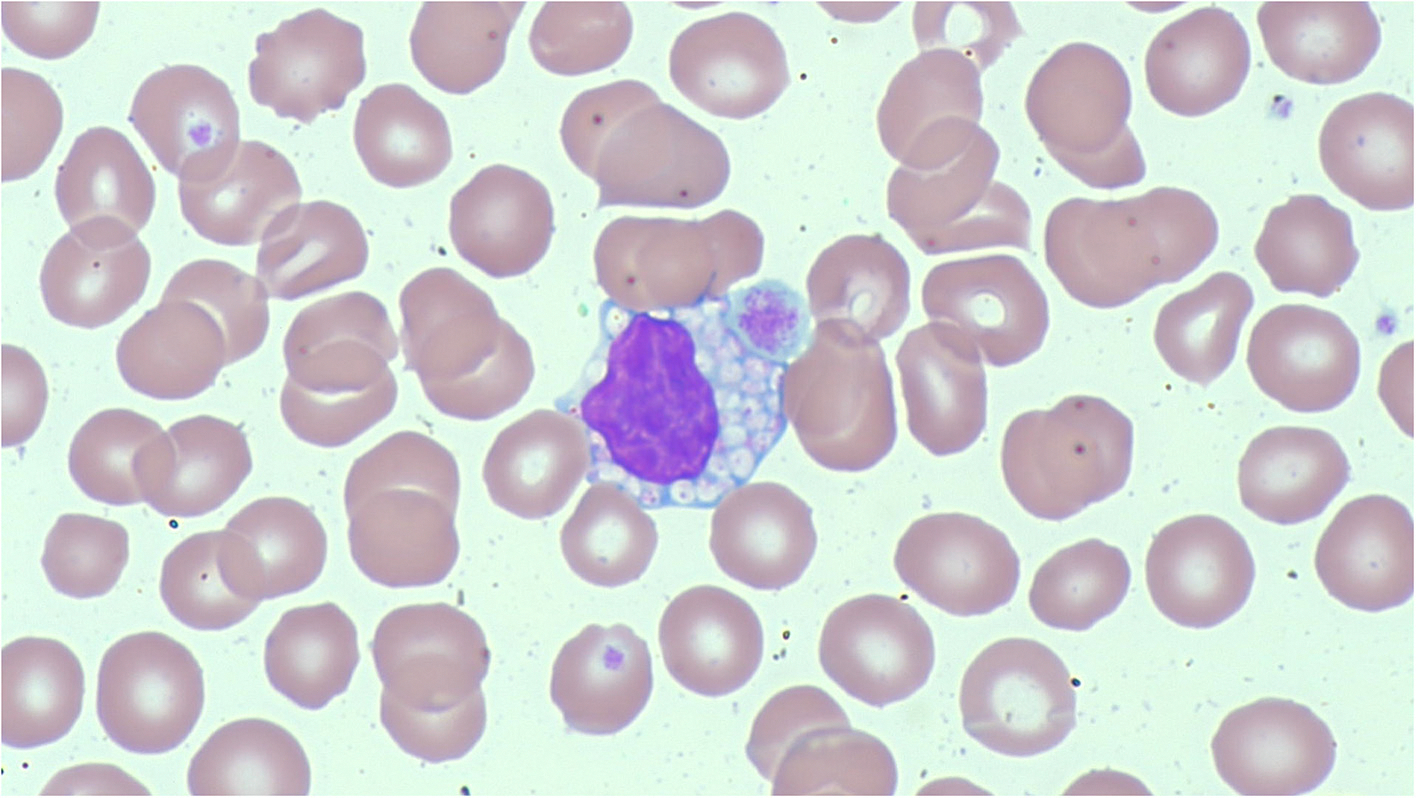
A vacuolated lymphocyte beside a large platelet, from a person with Mucolipidosis Type II (I-Cell disease). Of note, <10% of the lymphocytes in this blood film were vacuolated. (MGG, x60).

Vacuolation of lymphocytes are the typical blood film abnormality associated with metabolic disease, and are found in range of metabolic conditions, predominantly storage disorders (Fig. 3). The metabolic product responsible for the vacuolation depends on the underlying metabolic defect, and can be glycogen, lipid or mucopolysaccharide. An accumulation of these products in lymphocytes causes the abnormal appearance of the lymphocytes. Causes include CLN3 disease, Pompe disease, mucolipidosis type II, mucopolysaccharidoses, galactosialidosis, Niemann-Pick disease type A, Wolman disease, and GM1 gangliosidosis [18,19].

Vacuolation of white blood cells other than lymphocytes generally do not indicate a metabolic disorder. Normal monocytes are vacuolated in the resting state, and neutrophil vacuolation is a common finding in sepsis or inflammation, frequently accompanied by an increase in granules in the cytoplasm (termed “toxic granulation”). Jordan’s anomaly was originally described as a familial abnormality of white cell morphology, specifically abnormal vacuolation of neutrophils and neutrophil precursors, monocytes, eosinophils, basophils, and occasional lymphocytes. This can be found in the Chanarin-Dorfman syndrome (CGI-58 deficiency) and Adipose triglyceride lipase deficiency, which are both disorders of cytoplasmic triglyceride metabolism, and also referred to as Neutral lipid storage disease [18].

The Alder-Reilly phenomenon describes azurophilic lymphocyte inclusions surrounded by halos which can be seen in Tay-Sachs disease or mucopolysaccharidoses, in association with lymphocyte vacuolation [18].

The Pelger-Huet anomaly describes neutrophils with a reduction in the number of nuclear lobes present. Normal neutrophils have between 3 and 5 nuclear lobes, and Pelger-Huet neutrophils typically have bilobed nuclei, making them shaped like dumbbells or peanuts. The Pelger-Huet anomaly can be inherited in an autosomal dominant fashion due to variants in the Laminin B Receptor (*LBR*) gene where it is found without any other clinical sequelae. Acquired Pelger-Huet abnormality is commonly seen in myelodysplastic syndromes or in acute leukemia [18]. More recently, the Pelger-Huet abnormality has been described in NBAS deficiency [20] which may present to the metabolic physician given the nature of this recurrent acute liver failure syndrome which may involve multiple systems, and the finding of Pelgeroid neutrophils could aid in reaching a diagnosis.

Hypersegmented neutrophils (6 or more nuclear lobes) may be seen in megaloblastic anemia in both acquired forms and inherited forms, along with red cell changes described below.

#### 2.3.2. Red blood cell morphology

Inherited disorders of cobalamin or folate metabolism which cause megaloblastic anemia may present with a classical blood film appearance. In overt cases, the complete blood count (CBC) will show a macrocytic anemia, which progresses to pancytopenia. Hypersegmented neutrophils and oval shaped macrocytic red blood cells are the typical features on the blood film. There is usually strikingly abnormal red cell morphology, with marked variation in red blood cell size and shape due to the ineffective erythropoiesis [18]. Of note, these features may be subtle or absent in some cases. A markedly elevated LDH and bilirubin is usually seen, which can lead to confusion with hemolysis or malignancy. While disorders of absorption and transport of cobalamin and folate are classically associated with megaloblastic anemia, this is not a characteristic finding of many of the intracellular inherited cobalamin or folate disorders (e.g., Cbl A, B, G; MTHFR and others); therefore, the absence of megaloblastic anemia does not rule out all cobalamin or folate disorders.

**Fig. 4.**
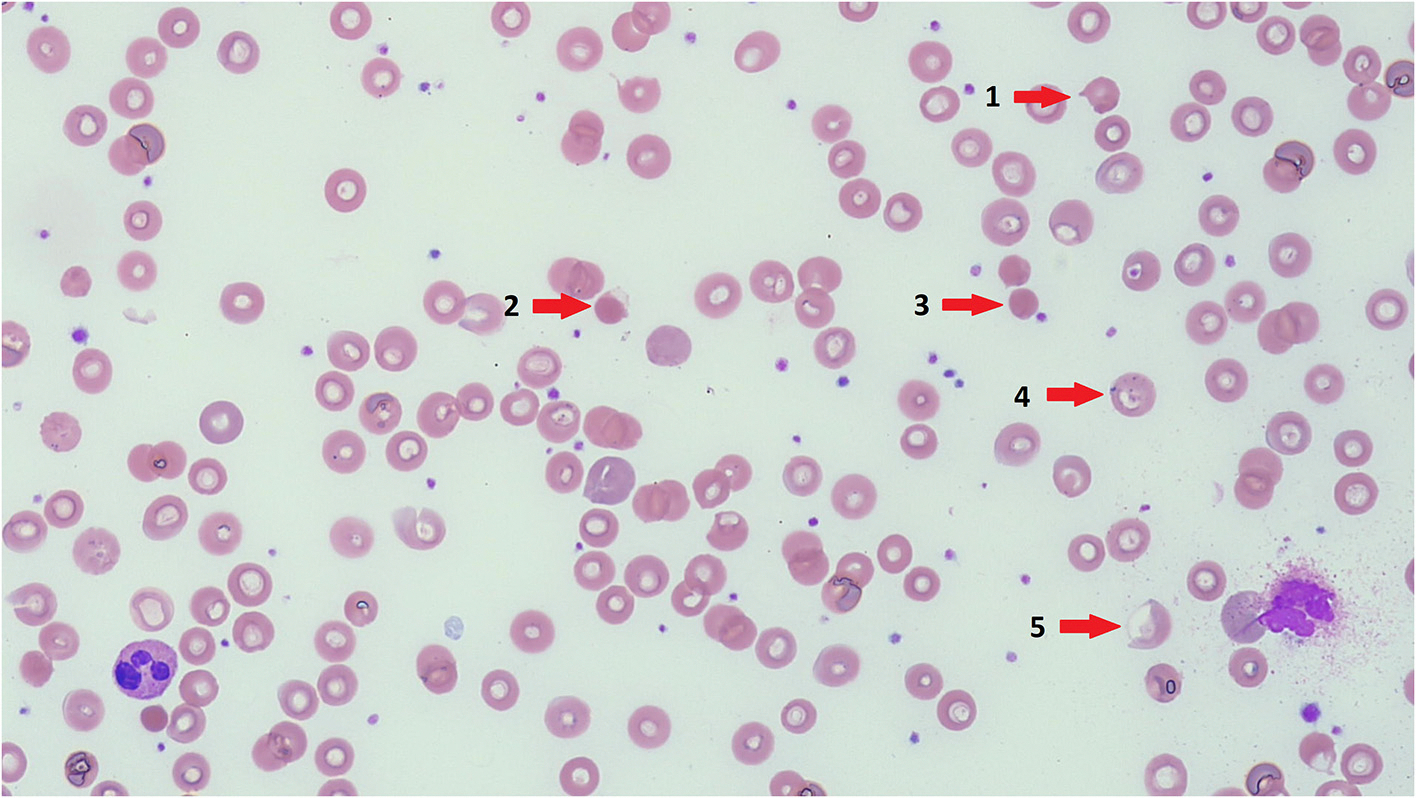
Blood film from an African child with G6PD deficiency, demonstrating polychromasia (red cells with a slight blue tinge), as well as many other red cell changes including 1) Keratocytes 2) Blister cells 3)Spherocyte 4) Basophilic stippling 5) Hemighost cell. (MGG x20). (For interpretation of the references to colour in this figure legend, the reader is referred to the web version of this article.)

Several IMDs are associated with hemolysis (see section). Hemolysis is suggested on blood film examination by the presence of anemia in combination with polychromasia. Polychromasia describes red cells that are reddish-blue due to the staining of both hemoglobin (red) and residual RNA (blue) (Fig. 4). These are the morphological equivalent of reticulocytes, and if there is polychromasia, the reticulocyte percentage will be elevated. Further examination of the red blood cell morphology will give clues as to the underlying cause of the hemolysis; for example, spherocytes in autoimmune hemolytic anemia or hereditary spherocytosis, or sickle cells in sickle cell disease. Acanthocytes are red cells with spiky projections of unequal length and distribution and are typically seen in association with hemolytic anemia in people with abetalipoproteinemia. They are also seen in hypobetaliproteinemia, and vitamin E deficiency [18]. Acanthocytes can also be seen in advanced liver disease, which is referred to as “spur cell” hemolytic anemia, for example in advanced Wilson disease or neonatal haemochromatosis. Stomatocytes are typically seen in sitosterolemia, with an associated hemolytic anemia, splenomegaly and abnormally large platelets [21]; they can also be seen in certain forms of GLUT1 deficiency syndrome [22].

#### 2.3.3. Bone marrow morphology

**Fig. 5.**
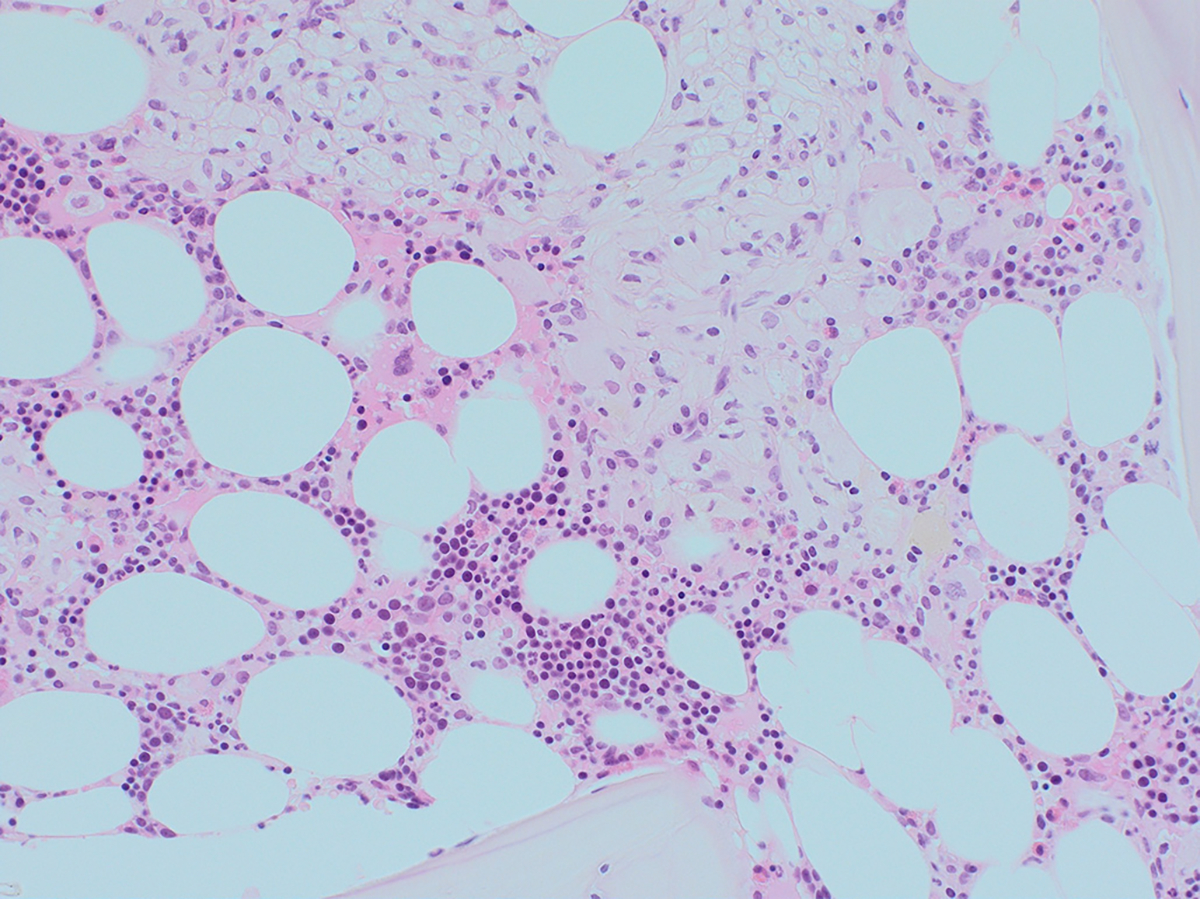
Gaucher Disease, bone marrow trephine section. There is a focal infiltrate of macrophages (top, centre) which have the typical “onion skin” or “watered silk” texture to the cytoplasm. This is due to the accumulation of glucosylceramide in the macrophage. The remainder of the trephine is comprised of normal hematopoietic tissue. (H&E, x200), Courtesy of Dr M Jeffers.

A total of 31 IMDs are listed in the IEMbase as having abnormal bone marrow findings. Gaucher disease is a lysosomal storage disorder caused by pathogenic variants in the *GBA1* gene leading to a deficiency in glucocerobrosidase, a lysosomal enzyme. This leads to accumulation of glucosylceramide in macrophages [23]. Type 1 Gaucher disease often presents to a hematologist, with splenomegaly and isolated thrombocytopenia due to hypersplenism. Over time, pancytopenia usually develops. These patients can be misdiagnosed as having immune thrombocytopenia, or even a lymphoproliferative disorder. In some cases, splenectomy is undertaken prior to the correct diagnosis. A high index of suspicion on behalf of the hematologist is required for accurate diagnosis, and measuring leucocyte beta-glucocerebrosidase should be part of the evaluation of people with unexplained splenomegaly, when more common causes have been excluded. The blood film may be unremarkable. The presence of “Gaucher Cells” on the bone marrow aspirate or trephine biopsy suggests the diagnosis (Fig. 5). There are usually increased numbers of abnormal macrophages in clumps or sheets, which are described as having an “onion skin” or a “watered silk” type appearance relating to the abnormal deposition of glucosylceramide [24]. These have a typical appearance but require a trained eye and a high index of suspicion.

Foamy macrophages describe large macrophages with lipid inclusions. These are classically seen in the bone marrow of people with Niemann-Pick disease type A. A number of other metabolic defects including familial hypercholesterolemia, Wolman disease/cholesteryl ester storage disease, Fabry disease and ceroid lipofuscinosis are also associated with foamy macrophages. These can also be seen in a variety of hematological and reactive conditions so are not specific for a metabolic disorder [24].

Sea blue histiocytes are distinctive macrophages containing ceroid or lipofuscin, and can be seen in Apolipoprotein E deficiency, Niemann-Pick disease (particularly type C) and Fabry disease, along with a variety of other hematological and reactive conditions, similar to foamy macrophages [24].

Bone marrow crystal deposition can be seen in cystinosis and hyperoxaluria (see pancytopenia section) [24].

Ringed sideroblasts describe red blood cell precursors in the bone marrow (erythroblasts) which have a ring of iron-laden mitochondria around the nucleus. These are only visible with a dedicated iron stain, e. g., Perl’s Prussian blue. They are seen in sideroblastic anemia (see section) [24].

### 2.4. Neutropenia

Twenty-seven IMDs in the IEM Knowledgebase are associated with neutropenia.

Barth syndrome is an inherited disorder of mitochondrial phospholipid metabolism, which is caused by variants in the *TAZ* gene, located on the X chromosome [25]. *TAZ* encodes Tafazzin, a membrane associated transacylase which is responsible for remodeling the inner mitochondrial membrane phospholipid, cardiolipin. It has an X-linked recessive pattern of inheritance. It leads to a variable clinical phenotype, characterized by cardiomyopathy, skeletal muscle myopathy, neutropenia, and constitutional growth delay. The neutropenia described in Barth syndrome is quite variable. In a case series of 88 affected males, neutropenia was noted in 84% [26]. The pattern of neutropenia varied from intermittent and unpredictable, cyclical neutropenia, to severe chronic neutropenia. The neutropenia is associated with an increased risk of severe bacterial infections, which can be ameliorated by treatment with granulocyte stimulating factor (G-CSF), and prophylactic antibiotics [26].

Severe neutropenia is seen in Glycogen Storage Disease Type 1b (*SLC7A4*), and is associated with recurrent bacterial infections and mouth ulcers [27]. G-CSF was the mainstay of treatment until quite recently. The use of a sodium-glucose co-transporter inhibitor, empagliflozin, has been found to be a very effective treatment for the neutropenia (and related complications), meaning G-CSF is often no longer needed in these patients [28]. Empagliflozin through inhibition of 1,5-anhydroglucitol (1,5-AG) renal reabsorption reduces the abnormal accumulation of toxic 1,5-AG6P in neutrophils in GSD1b, thus reducing neutrophil apoptosis. A similar effect is seen in patients with neutropenia due to G6PC3 deficiency.

**Fig. 6.**
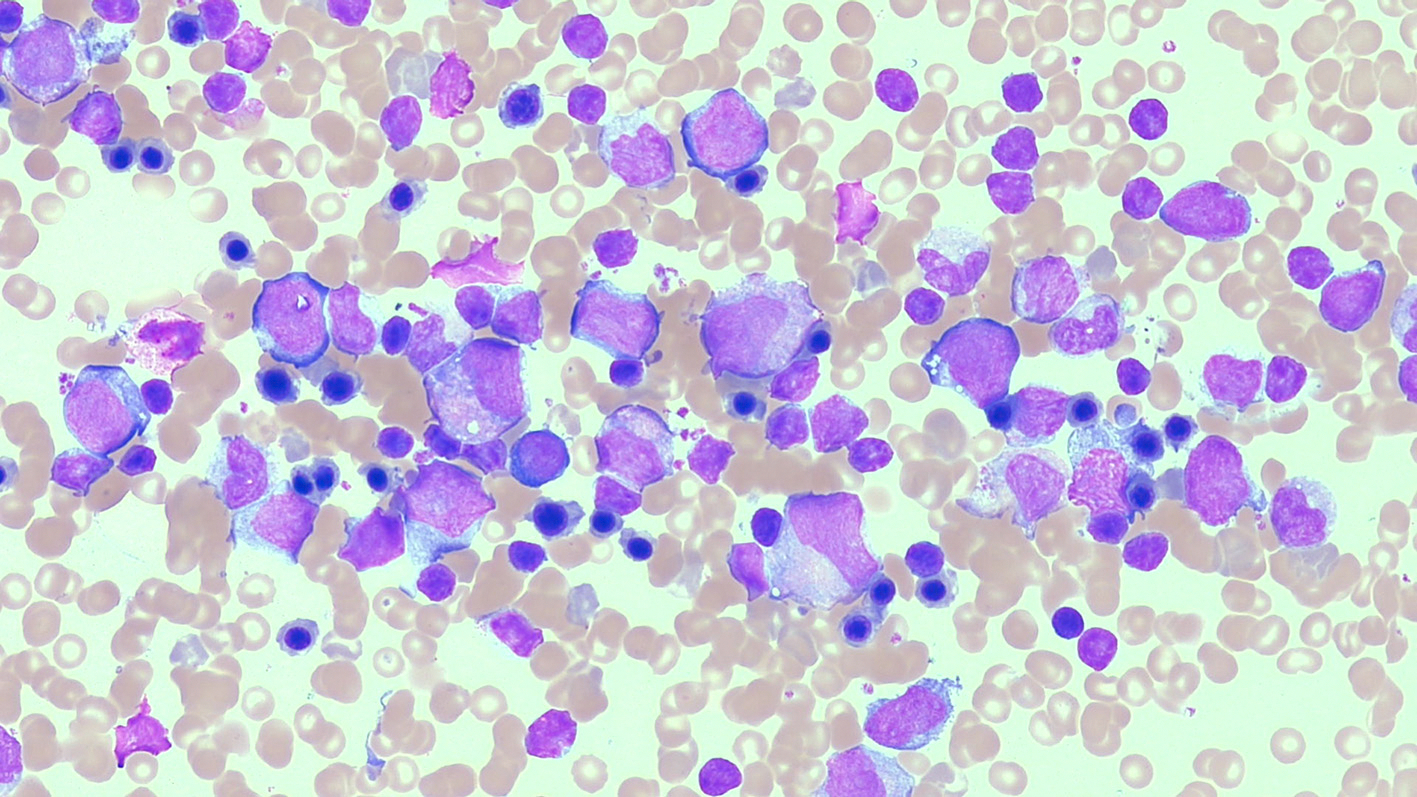
Bone marrow aspirate from a child with ALG13-CDG who presented with severe congenital neutropenia as part of his clinical phenotype, demonstrating complete maturation arrest of the granulocyte series, with only very immature granulocytic cells present, along with normal red cell maturation, and monocyte precursors. (MGG x40). (For interpretation of the references to colour in this figure legend, the reader is referred to the web version of this article.)

Several congenital disorders of glycosylation (CDGs) are associated with neutropenia (Fig. 6). Glycosylation is the post-translational modification of proteins by the addition of glycan chains. CDGs are an expanding group of about 170 IMDs which result in aberrant glycosylation of proteins or lipids, interfering with their function [29]. Because of this, CDGs have a wide range of symptoms including neurological symptoms, skeletal abnormalities, endocrine abnormalities, hepatic disease, cardiac and renal abnormalities, and coagulopathy. PGM3-CDG is associated with a severe immunodeficiency syndrome which includes neutropenia and has been treated with hematopoietic stem cell transplantation [30]. Cohen syndrome is caused by mutations in the *VPS13B* gene, which encodes a protein involved in glycosylation in the Golgi zone. Neutropenia is common, and often severe, and can lead to recurrent infections, aphthous ulcers and gingivitis, requiring G-CSF treatment [31]. JAGN1-CGD leads to a clinical picture of severe congenital neutropenia with maturation arrest in the bone marrow at the promyelocyte stage and is known to pediatric hematologists as a form of severe congenital neutropenia [32]. G6PC3-CDG is another cause of severe congenital neutropenia which is often accompanied by other anomalies including congenital heart defects, prominent superficial veins, urogenital anomalies, facial dysmorphism, growth and developmental delay and intermittent thrombocytopenia [28].

Shwachman-Diamond syndrome is a ribosomal biogenesis defect characterized by neutropenia/bone marrow failure, exocrine pancreatic insufficiency, and skeletal dysplasia. The majority of cases are caused by biallelic variants in the *SBDS* gene, and patients are typically looked after by hematologists.

### 2.5. Thrombocytopenia

A search of the IEMBase reveals 46 IMDs associated with thrombocytopenia. Gaucher disease can present with splenomegaly and thrombocytopenia and patients are frequently initially referred to a hematologist for evaluation. Thrombocytopenia is the most common hematological abnormality; however, it can progress to pancytopenia due to hypersplenism and bone marrow infiltration with Gaucher cells. Hypergammaglobulinemia may also be found as part of the hematological evaluation. Bone marrow aspirate and biopsy can suggest the diagnosis; however, these invasive tests are not a necessary part of the workup if the diagnosis is confirmed by measuring leucocyte β-glucocerebrosidase level and *GBA1* molecular testing. Treatment with enzyme replacement therapy ameliorates the hematological abnormalities [23]. There is an increased risk of hematological malignancies, particularly myeloma and lymphoma [33]. Niemann-Pick disease types A/B and C are also associated with thrombocytopenia and splenomegaly. The appearance of “foamy macrophages” on bone marrow aspirate and trephine biopsy suggest the diagnosis [24]. Several disorders including Hermansky-Pudlak syndrome and Gray Platelet Syndrome have long been considered inherited platelet disorders and cared for by hematologists are also considered IMDs due to their involvement in organelle biogenesis and vesicular trafficking, respectively.

### 2.6. Pancytopenia

Twenty-four IMDs in the IEM knowledgebase are associated with pancytopenia. The organic acidurias, methylmalonic aciduria (MMA), isovaleric aciduria (IVA) and propionic aciduria (PA), can all present with pancytopenia during the initial phase of metabolic acidosis [34]. This improves with good metabolic control. Gaucher disease can progress from isolated thrombocytopenia to pancytopenia due to bone marrow infiltration and hypersplenism, as can Niemann-Pick disease type A [24]. Mucolipidosis type II can present with pancytopenia in the neonatal phase which spontaneously improves (personal experience). The inherited disorders of cobalamin metabolism that lead to megaloblastic anemia including transcobalamin II deficiency and intrinsic factor deficiency can all present with a pancytopenia in combination with megaloblastic anemia [35]. Megaloblastic anemia can be diagnosed on a blood film with the combination of hypersegmented neutrophils and oval macrocytosis. There is often an unconjugated hyperbilirubinemia and a markedly elevated LDH which can lead to confusion with a hemolytic picture, but the reticulocyte count is not elevated [18]. Inherited disorders of folate metabolism such as dihydrofolate reductase deficiency and proton coupled folate transport deficiency present with similar hematological features [36].

The primary hyperoxalurias are a group of disorders of glyoxylate metabolism that cause overproduction of oxalate, which is usually excreted by the kidneys. High concentrations of oxalate lead to kidney stones, and deposition of calcium oxalate crystals in the kidney. The combination of postrenal obstruction and chronic tubulo-interstitial inflammation leads to renal failure in over 70% of patients. When the capacity of the kidneys to excrete oxalate is overwhelmed, there is systemic deposition of oxalate in the bone, heart, vessels, nerves, and eye. Bone marrow deposition of oxalate can lead to pancytopenia [37]. The typical appearance on the peripheral blood film is leucoerythroblastosis. A leucoerythroblastic blood film is where both erythroid and myeloid precursors, which are normally only present in the bone marrow, can be seen in the peripheral blood, usually with a typical “tear drop” appearance of red blood cells [18]. Bone marrow histology in these cases reveals deposition of oxalate crystals with surrounding granulomatous inflammation [24]. The primary hyperoxalurias can be treated with dietary restrictions, hyperhydration and pyridoxine in some cases. Renal replacement therapy may be required. Liver transplantation is potentially curative. RNAi therapy is a recent advance which could potentially markedly improve disease outcomes for patients while avoiding liver transplantation [38].

Griscelli syndrome is a rare autosomal recessive disorder caused by variants in the *RAB27A* gene, leading to hypopigmentation, slivery/gray hair and immunodeficiency. Pancytopenia can develop due to hemophagocytic lymphohistiocytosis [39].

### 2.7. Anemia

The most common cause of anemia is iron deficiency, which can affect any patient with inadequate dietary intake of iron, or reduced iron absorption. CBC will typically reveal a hypochromic, microcytic anemia as evidenced by a reduced mean corpuscular hemoglobin (MCH) and mean corpuscular volume (MCV), respectively. Iron deficiency can be confirmed with a reduced serum ferritin. However, it should be noted that ferritin levels are often artificially elevated in the presence of systemic inflammation or kidney disease, so a normal or even elevated serum ferritin does not exclude iron deficiency. In this setting, iron studies can provide further information, but are often susceptible to similar changes in the setting of inflammation [40].

Anemia of chronic disease can occur in any disorder where there is co-existent chronic inflammation. In a study of hematological abnormalities in 319 patients with IMDs in Turkey, anemia of chronic disease was the most common hematological finding encountered [41]. This is usually characterized by a normocytic, normochromic anemia, and is a diagnosis of exclusion. The pathogenesis of anemia of chronic disease involves iron-restricted erythropoiesis due to upregulation of hepcidin. Increased red cell turnover and reduced production of erythropoietin also play a role in the pathogenesis. Iron deficiency anemia frequently co-exists with anemia of chronic disease, and it can be very challenging to diagnose in this setting, as the serum ferritin is unreliable. Novel red cell indices available on modern automated blood count analyzer such as the reticulocyte hemoglobin concentration (RET–He) and the percentage of hypochromic reticulocytes (%HYPO) can predict functional iron deficiency where the ferritin is unreliable, and predict response to iron replacement [42].

Anemia is very common in people with glycogen storage disease type 1, and is frequently multifactorial with causes including iron deficiency, renal impairment, anemia of chronic disease and hepatic adenomas [27]. Iron deficiency is particularly common, possibly due to the restrictive nature of the diet, along with increased losses due to bleeding diathesis. Some patients with hepatic adenomas were observed to have severe anemia refractory to oral iron therapy. Increased hepcidin expression was demonstrated in hepatic adenoma tissue, and one patient experienced resolution of anemia with resection of the adenoma. It was postulated that aberrant hepcidin expression from the hepatic adenomas was the cause of the anemia [43].

Iron-refractory iron deficiency anemia (IRIDA) is a rare autosomal recessive disorder caused by loss-of-function variants in the *TMPRSS6* gene, leading to a deficiency in an enzyme called Matriptase-2. This results in up-regulation of hepcidin which is a key regulator of iron homeostasis, leading to a clinical picture of iron deficiency which responds poorly to oral iron. The condition can be treated with intravenous iron supplementation [44].

Hemolytic anemia can present with a normocytic or macrocytic anemia. An elevated reticulocyte count or blood film examination showing polychromasia is often the first clue towards a hemolytic anemia. An elevated LDH and unconjugated hyperbilirubinemia provide further evidence of hemolysis, as does a low haptoglobin, although it should be noted that haptoglobin is unreliable in the first year of life and in liver disease [45].

Of the inherited causes of hemolytic anemia, disorders of the red cell membrane and hemoglobinopathies are most commonly implicated. Disorders of red cell enzymes (commonly termed “enzymopathies”) are also an important cause of hereditary hemolytic anemia; patients with these IMDs are predominantly looked after by hematologists. Mature red cells contain no mitochondria and are totally dependent on anaerobic glycolysis and the pentose phosphate pathway for their energy needs. Deficiencies of enzymes within these pathways can cause a hemolytic anemia as a result of deficiency of ATP or reducing equivalents. The diagnosis of these disorders is often suggested by the clinical features and blood film examination; however, exact diagnosis usually requires measuring the enzyme in question, or more commonly now, genetics panels using next generation sequencing (NGS).

By far the most common enzyme deficiency causing hemolysis is glucose-6-phosphate dehydrogenase (G6PD) deficiency (Fig. 4). This occurs at high frequency in Mediterranean, African and Asian populations. It is an X-linked recessive disorder of variable severity, characterized by acute episodes of hemolysis brought on by the ingestion of oxidizing drugs or fava beans (broad beans). Patients typically present with dark “cola” or tea-colored urine due to hemoglobinuria, along with acute onset symptomatic anemia. Neonatal jaundice is common, often without anemia. It should be noted that G6PD levels measured during the acute hemolytic phase are frequently falsely normal in the presence of reticulocytosis, because of a higher concentration of G6PD in reticulocytes compared to mature red blood cells. Management is supportive in the acute phase with folic acid supplementation and transfusion support if necessary, and in the long term requires avoidance of oxidizing medications and fava beans. Rarely, G6PD deficiency can lead to a chronic hemolytic anemia with acute exacerbations by oxidizing substances [46].

Pyruvate kinase deficiency is the most common red cell enzymopathy affecting Caucasian people. As with any chronic hemolytic anemia, there is associated splenomegaly, jaundice, hyperbilirubinemia and a propensity towards developing gallstones [47]. Other hereditary deficiencies of enzymes involved in anaerobic metabolism are extremely rare, and are mostly associated with other congenital abnormalities such as learning difficulties (hexokinase, glucose phosphate isomerase) or myopathy (phosphofructokinase/Glycogen Storage Disease type VII) [18].

Hemolytic anemia can be seen in advanced liver disease due to metabolic disorders, where it is termed “spur cell anemia” due to the presence of acanthocytes on the blood film. Hemolytic anemia can be seen in the organic acidurias MMA, PA and IVA. Abetaliproproteinemia and sitosterolemia are two disorders of lipid metabolism associated with hemolytic anemia, with acanthocytes and stomatocytes seen on the blood film respectively [18]. Cobalamin C disease is a rare cause of microangiopathic hemolytic anemia, which presents as an atypical hemolytic uremic syndrome (aHUS), with hemolysis and renal failure due to thrombotic microangiopathy of the renal microvasculature [48]. Plasma homocysteine and methylmalonic acid levels are typically high, and treatment includes B12 injections and folate.

Megaloblastic anemia is characterized by macrocytic anemia, with the presence of megaloblasts on bone marrow biopsy. It is most often caused by B12 or folic acid deficiency, due to malabsorption or inadequate diet. Inherited disorders of cobalamin metabolism that lead to megaloblastic anemia in infancy or early childhood include transcobalamin II deficiency, Imerslund-Grasbeck syndrome and gastric intrinsic factor deficiency [35]. Megaloblastic anemia can be diagnosed on a blood film with the combination of hypersegmented neutrophils and oval macrocytosis. There is often an unconjugated hyperbilirubinemia and a markedly elevated LDH which can lead to confusion with a hemolytic picture, but the reticulocyte count is not elevated. Inherited disorders of folate metabolism such as dihydrofolate reductase deficiency and proton coupled folate transport deficiency present with similar hematological features. Most cases of megaloblastic anemia, even in childhood, are due to deficiency of either vitamin B12 or folate, due to malabsorption (as in pernicious anemia) or diet. In the case of breastfed infants, if the mother is deficient in vitamin B12, the infant may also be at risk of deficiency. However, a high index of suspicion is required for those presenting at a young age. Investigations should include serum total vitamin B12, active vitamin B12 (holotranscobalamin), serum and red cell folic acid level, total homocysteine, urinary organic acids including methylmalonic and orotic acid, amino acids, +/− methylmalonic acid. It is increasingly recognized that total serum vitamin B12 levels are neither sensitive or specific, and measuring active vitamin B12 (holotranscobalamin) is far more sensitive and specific [49]. In transcobalamin II deficiency the serum vitamin B12 is normal, but because there is an intracellular deficiency of cobalamin, homocysteine and methylmalonic acid are raised. In Imerslund-Grasbeck syndrome and gastric intrinsic factor deficiency, the serum vitamin B12 is low, because the defect is in the absorption of B12; homocysteine and methylmalonic acid are raised.

The sideroblastic anemias are characterized by the presence of cells called ringed sideroblasts, which are seen on bone marrow aspirates using a specific iron stain such as Perl’s stain [24]. These are nucleated red blood cell precursors which have a ring of iron-laden mitochondria around the nucleus. Depending on the underlying cause of the sideroblastic anemia, the CBC may reveal microcytic, normocytic or macrocytic red cells, or a dimorphic red cell picture may be present. The most common form of sideroblastic anemia is an acquired condition as part of a myelodysplastic syndrome, which is a malignant condition of the bone marrow where acquired clonal variants lead to ineffective hematopoiesis and cytopenia. Acquired sideroblastic anemia is also seen in lead poisoning, copper deficiency (which may be secondary to zinc toxicity), and due to medications including chloramphenicol, isoniazid, and linezolid. The congenital sideroblastic anemias include Pearson syndrome, Kearns-Sayre syndrome, X-linked sideroblastic anemia due to pathogenic variants in delta-aminolevulinate synthase 2 (which may be responsive to pyridoxine supplementation), and thiamine-responsive megaloblastic anemia. Pearson syndrome is a rare mitochondrial disorder caused by mitochondrial DNA deletions. It usually presents in infancy with severe hyporegenerative anemia, associated with exocrine pancreatic insufficiency. Other manifestations include failure to thrive, lactic acidosis, muscle hypotonia, renal tubulopathy and cardiac dysfunction. The anemia often spontaneously resolves in the first 1–3 years of life, but the overall prognosis is quite poor [50,51].

Some of the aminoacyl-tRNA synthetase (ARS) disorders such as MARS1 and LARS1 deficiencies are associated with chronic hypochromic microcytic anemia which may be transfusion dependent for periods of infancy and does not respond to iron [52,53]. These are disorders of protein synthesis affecting multiple systems and the exact mechanism behind the hematological features is as yet unclear.

Rarely IMDs can present with hydrops fetalis due to profound antenatal anemia and therefore it is important to consider metabolic disorders such as the lysosomal storage disorders (classically Sly disease) where nonimmune hydrops is present [54].

### 2.8. Coagulation abnormalities

Several groups of metabolic disorders are associated with a bleeding tendency. Coagulopathy due to severe liver disease can be seen in any metabolic disorder which progresses to either acute or chronic liver failure. The coagulopathy of liver disease is complex [55]. From a laboratory perspective, it is associated with prolongation of both the prothrombin time (PT) and the activated partial thromboplastin time (APTT), due to reduced levels of coagulation factors, most which are synthesized in the liver. The fibrinogen level is frequently low, due to decreased hepatic production or hyperfibrinolysis. The platelet count can be reduced, due to hypersplenism from portal hypertension, reduced liver production of thrombopoietin, or multifactorial in acutely unwell patients. The risk of bleeding is not proportionate to the degree of laboratory abnormalities. Indeed, there is increasing recognition that chronic liver failure is associated with an increased risk of thrombosis. This is thought to be the concept of “rebalanced hemostasis” where similar reductions in the levels of the natural anticoagulants, and elevated levels of factor VIII and von Willebrand factor counteract the reduced levels of other coagulation factors [55].

Vitamin K deficiency can occur in any IMD due inadequate dietary intake or prolonged antibiotic therapy. Vitamin K deficiency should be suspected in any patient with a significantly prolonged PT +/− APTT and treatment is usually started empirically. Response to treatment is considered a diagnostic test, as measuring vitamin K levels or PIVKAs (Proteins Induced by Vitamin K Absence) is expensive and not routinely available and is usually unnecessary. Several metabolic disorders are associated with malabsorption of fat-soluble vitamins, particularly those associated with intrahepatic cholestasis and abetalipoproteinemia [56]. There are also extremely rare inherited disorders of Vitamin K metabolism, caused by biallelic pathogenic variants of either gamma-glutamyl carboxylase or vitamin K2,3-epoxide reductase complex. These proteins are necessary for gamma carboxylation, which is a post-translational modification required for the function of the vitamin K dependent coagulation factors. This leads to variable reductions in the vitamin K-dependent clotting factors II, VII, IX and X, as well as the natural anticoagulants proteins C, S and Z. The spectrum of bleeding abnormalities varies from mild to severe. Developmental and skeletal abnormalities are also present which mimic fetal warfarin syndrome. Treatment can be with Vitamin K replacement which is variably effective, and plasma or four-factor prothrombin complex concentrate replacement for major bleeding or surgery. These disorders are comprehensively reviewed elsewhere [57].

Classical galactosemia is associated with a coagulopathy in the neonatal period which is out of proportion to the degree of liver impairment. In a large registry-based study, coagulopathy was noted in 42.5% of affected neonates [58]. This is thought to be partly due to liver impairment, but also due to abnormal glycosylation of coagulation factors. The PT and APTT are typically both prolonged, again out of proportion to the degree of liver impairment. There are case reports of retinal and vitreal hemorrhages related to local factors as well as the coagulopathy. If treatment is required for bleeding complications, plasma would be the treatment of choice. The coagulopathy resolves quickly once a galactose-free diet is established.

In glycogen storage disease type 1, there is a bleeding diathesis characterized by mucocutaneous bleeding such as easy bruising, epistaxis, menorrhagia [27]. Abnormalities of platelet function have been described, which appear to be ameliorated by dietary intervention and good metabolic control [59]. It is hypothesized that there is abnormal glycogen deposition in the platelets which leads to an acquired dysfunction. A von Willebrand disease-like defect has also been described, with either reduced von Willebrand factor levels or dysfunction [27]. CBC, coagulation screen, von Willebrand antigen and functional testing as well as platelet function tests should be included as part of the evaluation if there are bleeding symptoms. Standard treatment options for bleeding associated with platelet function defects or von Willebrand disease include tranexamic acid, desmopressin, platelet transfusion or von Willebrand factor concentrate. There is no specific evidence to guide treatment in this patient population, although there are small case series describing successful use of desmopressin in people with glycogen storage disease type 1a [60,61]. Treatment for bleeding symptoms should be directed by an expert in hemostasis. Caution has been advised with desmopressin due to the risk of fluid overload and hyponatremia in the setting of IV glucose administration [27].

CDGs are variably associated with a coagulopathy [62]. Coagulation factors are highly glycosylated proteins, which explains why there are laboratory and clinical features of coagulopathy affecting many patients with congenital disorders of glycosylation. Many disorders of N-linked glycosylation appear to cause a reasonably similar pattern of coagulation abnormalities, characterized by a reduction in the levels of Factor XI, Antithrombin III (AT III), Protein S (PS) and Protein C (PC). PMM2-CDG is the most common form of CDG, and the coagulopathy is the best characterized [63]. Clinically, it is characterized by a tendency towards thrombotic events rather than bleeding, presumably due to the reduced levels of natural anticoagulants, AT III, PC, PS. It is not understood why abnormal glycosylation leads to the reduction in these factors particularly. Glycosylation of AT III is the best studied, and it is thought that abnormal glycosylation reduces protein secretion as well as enhancing clearance [62]. In a systematic literature review, 12.5% of patients with PMM2-CDG were found to have experienced thrombotic events [64]. There is evidence that the coagulopathy worsens during times of acute illness [63]. There are no evidence-based guidelines to support decision making; however, consideration should be given towards thromboprophylaxis during high-risk periods after consultation with an expert in thrombosis and hemostasis. It is important to realize that these disorders are ultra-rare, and it is therefore not possible to provide definitive conclusions as to how abnormal glycosylation of coagulation factors will affect an individual patient. It is advisable to seek expert advice from a hematologist, and measure coagulation factors to determine the likely risk of thrombosis or bleeding for each individual patient. Hemostatic abnormalities in CDG have been comprehensively reviewed elsewhere [62].

Classical homocystinuria (HCU) due to cystathionine beta-synthase (CBS) deficiency is the typical metabolic disorder associated with a hypercoagulable state. Left untreated there is a 30% chance of a thromboembolic event by age 20, rising to 50% by age 30. Thromboembolic events can be either arterial or venous. In the largest case series of untreated patients, venous thromboembolic disease was the most common manifestation (50%) followed by stroke which included cerebral venous sinus thrombosis (32%). Peripheral arterial disease accounted for 11% and myocardial infarction for 4% [65]. The cornerstone of prevention is treatment of the underlying illness with pyridoxine and dietary management to maintain a plasma total homocysteine level of <100 μmol/L [66]. There is lack of an evidence base to suggest interventions to reduce the risk of thrombosis; however, suggestions can be extrapolated from other high-risk conditions, such as inherited thrombophilia. Consideration should be given to thrombosis risk and thromboprophylaxis during high-risk periods, for example, surgical procedures, immobilization, and intercurrent illness. Patients should be kept well hydrated, with early recourse to intravenous fluids for example in the setting of vomiting or diarrhea. All people admitted to hospital should have a risk assessment for venous thromboembolism (VTE), and consideration be given to pharmacological and mechanical thromboprophylaxis. Early and regular mobilization should be encouraged after any surgical procedure. Female patients should avoid estrogen-containing contraception, or other estrogen-containing medication such as hormone replacement therapy (58). Pregnancy is a high risk period for thrombosis [67], and women should be referred to a hematologist with a special interest in obstetric hematology for guidance on thromboprophylaxis. The 2019 consensus guidelines for the management of classical HCU suggest that all women should receive LMWH thromboprophylaxis during the third trimester of pregnancy and for 6 weeks post-partum. Women with additional risk factors should receive thromboprophylaxis throughout the pregnancy [66]. Case reports of successful pregnancies in women with non-pyridoxine responsive classical HCU highlight the complex nature of pregnancy in these conditions, and the high risk of thrombosis, with one woman developing a pulmonary embolism at 11 weeks postpartum, having stopped prophylactic low molecular weight heparin just 2 weeks postpartum [67].

Mild elevation of homocysteine found in association with the common *MTFHR* polymorphism 677C > T occurs in 5–7% of the population. While it was previously thought that there was an association of this *MTHFR* variant with VTE, subsequent evidence has shown it is not associated with venous thrombosis [68]. Rarer severe deficiency of MTFHR may cause severe hyperhomocystinemia (>100 μmol/L) and is associated with neurodisability (due to CSF folate deficiency) and hypercoagulation [69].

### 2.9. Diagnosis and differential diagnosis

Initial hematological investigations should begin with a full blood count and a blood film, preferably examined by an experienced hematologist. Providing adequate clinical details is critical for optimal results from blood film examination. Further hematological investigations should be directed by the presenting symptom(s). The first step in exploring a possible bleeding tendency should be a structured clinical history using a standardized questionnaire, for example, the Bleeding Assessment Tool (BAT) as developed by the international Society of Thrombosis and Hemostasis [70,71]. Further investigations should be directed by the results of the BAT score. It should be noted that the BAT score may not be as useful in children, and pediatric-specific bleeding assessment tools have been developed [72]. An abnormal BAT score should be followed by measuring a PT, APTT, fibrinogen and von Willebrand screen as an initial step. If the PT or APTT is prolonged, the relevant coagulation factors can be measured. It should be noted that a normal PT or APTT does not exclude mild coagulation factor deficiencies, so consideration should be given to measuring coagulation factors regardless of the PT and APTT results. If clinical suspicion of a bleeding tendency persists despite normal tests as outlined above, the next step is to measure Factor XIII and perform platelet function tests by light transmission aggregometry, along with platelet nucleotide levels [73]. It should be noted that coagulation tests are affected by many pre-analytical and analytical variables [74]. They require a significant degree of expertise for interpretation, and investigation of a bleeding tendency should be directed by a hematologist.

The investigations of cytopenias should begin with a clinical history and examination of the blood film. The most common cause of transient neutropenia is a viral infection, and this should always be considered in metabolic patients with new-onset neutropenia. The neutropenia in these cases is often mild, and not associated with an increased risk of infection. In some cases, it can take several months to resolve. Medications, for example, antibiotics and anticonvulsant medications are other common causes of neutropenia, and may also cause thrombocytopenia, and should be considered as part of the evaluation. A polymorphism of the Duffy blood group gene (*ACKR1*) which causes the Duffy null red blood cell phenotype (Fya-b-) is very common in people of African descent and is associated with lower neutrophil ranges compared with White individuals. This has commonly been referred to as “benign ethnic neutropenia” but alternative names have been suggested. The Duffy receptor is used by *Plasmodium ovale* to enter the red blood cell, and the Duffy null phenotype confers a resistance to this type of malaria. The Duffy antigen is postulated to be a cytokine sink that binds to inflammatory cytokines, and therefore, there are relatively less neutrophils circulating in the blood stream. However, the total body neutrophil count is thought to be normal, and these people have no propensity towards infections [75].

Thrombocytopenia is frequently seen in severe sepsis and liver disease, and can also be due to medications, particularly anti-epileptic medications. If a new thrombocytopenia is identified, the hematology laboratory must first exclude pseudothrombocytopenia, which occurs when an erroneously low platelet count is recorded due to platelet clumping in the sample tube. This is related to the use of EDTA in the collection tube, is easily identified by blood film examination, and recurrent issues can be overcome by using citrate anticoagulant instead of EDTA [18].

Following exclusion of the more common causes of cytopenias and review of the blood film, useful initial investigations include biochemistry panels, LDH, ferritin, vitamin B12, and folate levels. In some instances, when a cause is not clear, a bone marrow aspirate and trephine biopsy are required to further evaluate cytopenias.

There has been a major revolution in diagnosing inherited hematological and IMDs with the advent of increasingly accessible next-generation sequencing (NGS) techniques such as gene panels and whole exome sequencing. This has made it much easier to accurately diagnose congenital disorders, for example, inherited platelet disorders and inherited neutropenic disorders as well as new and ultra-rare IMDs such as the CDGs.

### 2.10. Treatment

There is a lack of evidence-based guidance to direct the management of hematological complications of metabolic disease. Close collaboration between the metabolic physician and hematologist is required given the complex nature and rarity of these disorders. Many hematological complications of IMDs will resolve with adequate treatment of the underlying metabolic condition, such as enzyme replacement therapy for Gaucher disease, or homocysteine lowering treatments in classical HCU. Specific management of certain hematological complications are discussed below. With the current developments in the area of gene therapies, it is very likely these will provide future treatment options for some metabolic hematological disorders.

#### 2.10.1. Anemia

The underlying cause of the anemia in IMDs is key to treatment. Optimizing metabolic treatment and diet are key management strategies. Close working relationships between the metabolic pediatrician and hematologist are important for complex cases.

In anemia of chronic disease, accurately determining iron status predicts response to intravenous iron supplementation and is therefore key to appropriate management. Oral iron is often poorly absorbed in people with anemia of chronic disease due to inflammation-mediated upregulation of hepcidin, so intravenous iron is often necessary [40]. Blood transfusion support is occasionally required as a last resort in the setting of symptomatic anemia.

#### 2.10.2. Neutropenia

The link between neutropenia and severe infection is not necessarily clear. Guidelines for the management of febrile neutropenia are derived from chemotherapy-induced neutropenia; however, clinical experience would suggest that neutropenia due to other causes does not behave in the same way [76]. Neutropenia can be graded as mild (neutrophils 1–1.5 × 10^9^/L) moderate (0.5–1 × 10^9^/L) or severe (<0.5 × 10^9^/L). Prompt assessment and treatment of infections is the cornerstone of management for people with neutropenia. However, in many people with mild or moderate neutropenia, this may not be necessary. Doctors treating patients with febrile neutropenia should be aware of the risk of atypical infections such as fungal infections in these patients, particularly with concomitant steroid therapy, although again, any data for this comes from people undergoing treatment for hematological malignancy [77]. Patients and caregivers should be educated about the risk of overwhelming sepsis and advised to seek prompt medical attention in the case of fever.

In some people, granulocyte colony-stimulating factor (G-CSF) is indicated to bring up the neutrophil count. This is particularly useful in people with a history of recurrent bacterial infections, people with severe neutropenia, or in specific conditions that are known to be associated with infectious complications, for example, Barth syndrome [26]. Its use should be considered on a case-by-case basis and be directed by a hematologist. Side effects include bony pain, splenomegaly, and very rarely, splenic rupture. Prophylactic antibiotics can also be useful on a case-by-case basis. There is a theoretical concern that the chronic administration of G-CSF could lead to an increased risk of myelodysplastic syndrome or acute myeloid leukemia. However, there has never been any definitive evidence to support this hypothesis. An increased risk of transformation to myeloid malignancies has been demonstrated in patients with congenital neutropenia regardless of G-CSF administration, which makes drawing evidence-based conclusions regarding causality difficult. Furthermore, there is no increased risk of myeloid malignancy with G-CSF administration in people with autoimmune or idiopathic neutropenia, or in peripheral blood stem cell healthy donors. Future well designed clinical studies will hopefully provide more clarity on this important question [78,79].

Empagliflozin, an SGLT2 inhibitor, has revolutionized the management of people with glycogen storage disease type 1b, with improvement in neutrophil counts meaning that G-CSF is often no longer needed for these patients [28].

#### 2.10.3. Hypercoagulability

Despite the lack of evidence-based guidelines for the management of people with IMDs resulting in a prothrombotic tendency, we can extrapolate some commonsense recommendations from other thrombophilic states. As for other complications of metabolic illness, treatment of the underlying illness, particularly in classical HCU, will reduce the risk of thrombosis.

In patients with inherited thrombophilias, thrombosis often occurs during high-risk periods [80]. Although there is limited evidence, one would expect similar findings in IMDs with hypercoagulability, and attention to preventative measures during high-risk periods would be expected to reduce the rate of thrombosis. Surgical procedures, hospital admission for acute illness, immobility (including lower limb casting etc.), pregnancy, estrogen-containing medications (i.e., contraceptives and hormone replacement therapy) and to a lesser extent, long-haul travel are all considered high risk periods for thrombosis. On admission to a hospital, all patients should be assessed for their risks of VTE, and mechanical and/or pharmacological thromboprophylaxis be considered [81]. Early mobilization, prompt treatment of infections, and attention to hydration status during intercurrent illness are all important in reducing the risk of in-hospital thrombosis. In HCU, optimizing metabolic treatment and homocysteine levels reduces the risk of thrombosis [66].

A long-haul flight is generally accepted as a flight duration of >4 h. It is estimated that the relative risk of thrombosis during and immediately after a long-haul flight is doubled, which translates into only a very minor increase in the absolute risk of thrombosis. Nonetheless, the risk of VTE related to air travel is widely publicized, and very much in the public eye, making it a common question asked of hematologists. In order to reduce the risk of VTE, people with thrombophilia should be advised to stay mobile during the flight, stay well hydrated, avoid alcohol and sedative drugs, and wear compression stockings. Pharmacological thromboprophylaxis is very rarely indicated [82].

It is particularly worth mentioning that pediatric VTE is becoming increasingly common. The vast majority of pediatric VTE affects hospitalized children with underlying medical illness. Evidence suggests that children with IMDs make up a proportion of this, for example in a large study in the US, children with metabolic disorders made up 17% of children with a thrombotic event [83]. Typically regarded as a disease of adults, there may be less awareness among pediatricians of VTE, and there is little evidence base on which to make recommendations. Ideally, each pediatric hospital should have a thromboprophylaxis risk assessment guideline to direct thromboprophylaxis, as this has been shown to greatly improve the rates of thromboprophylaxis provided to high-risk patients [84].

The direct oral anticoagulants (DOACs) have revolutionized the management of venous thrombosis and have largely replaced warfarin anticoagulation as they are at least equally effective, do not require regular monitoring, and carry a lower risk of bleeding [84]. When deciding about the duration of anticoagulation, the most important factor to consider is whether the event is considered provoked or unprovoked [85]. Typically, people who have experienced VTE should receive a minimum of 3 months of anticoagulation [86]. The risk of recurrent VTE after a provoked event (e.g., after major surgery) is much lower than after an unprovoked event. For this reason, the majority of people can safely discontinue anticoagulation after a clearly provoked event, after a minimum of 3 months of treatment [84,86]. This recommendation generally includes people with inherited thrombophilia [86,87]. If the VTE is unprovoked, indefinite anticoagulation is generally indicated. There is little evidence base on which to provide specific recommendations for people with IMDs. Decisions regarding the duration of anticoagulation should involve a hematologist and consider the circumstances and site and extent of thrombosis, the risk of recurrence, the bleeding risk of the individual patient, lifestyle factors, the quality of metabolic control (in the case of classical HCU) and the patient’s preferences and values.

#### 2.10.4. Bleeding tendency

Treatment of bleeding in people with a bleeding tendency should be directed by the underlying hemostatic defect present. The possibility of vitamin K deficiency should always be considered in people with an abnormal coagulation screen and risk factors for vitamin K deficiency such as dietary, prolonged antibiotic use or malabsorption of fat-soluble vitamins. Treatment with vitamin K by the oral or IV routes can be given empirically. For other hemostatic abnormalities, e.g., the presence of a prolonged PT +/− APTT, treatment is only indicated in the presence of bleeding or in preparation for an invasive procedure. Tranexamic acid is a useful adjunct for the management of mucosal bleeding, for example epistaxis or menorrhagia. It is also very helpful in the prevention of surgical bleeding in people with a bleeding tendency, and in many mild bleeding disorders, is adequate for the majority of surgical procedures. However, it is worth noting that tranexamic acid can rarely be associated with seizures, most reports being associated with patients undergoing cardiac surgery. Occasionally, the use of blood products such as plasma, platelet transfusion, or specific coagulation factor concentrates is required, under guidance from a hematologist.

## 3. Conclusion

There is a significant interplay between metabolic disorders and hematology. Hematologists can be of assistance in diagnosing metabolic disorders and can aid with investigating and managing the hematological complications of metabolic disorders. It is important that hematologists are aware of the possibility that metabolic disorders can present with primarily hematological issues, to ensure timely and accurate diagnosis. Multidisciplinary management and co-operation will improve the diagnosis and management of these rare and challenging disorders. This represents the 16th issue in a series of educational summaries providing a comprehensive and updated list of metabolic differential diagnoses according to system involvement. The full list can be freely accessed at www.iembase.org/gamuts and will be curated and updated on a regular basis.

## Supplementary Material

1

2

## Figures and Tables

**Table 1:** Biochemical investigations in metabolic diseases presenting with hematological phenotype.

Basic tests	Profiles	Other tests

Full blood count	Amino acids incl. homocysteine (P)	Iron (S)
Blood film	Organic acids (U)	Transferrin (S)
Reticulocytes (B)	Acylcarnitines (DBS, P)	Ferritin (S)
ASAT/ALAT (P)	Sialotransferrins (S)	Copper (S, U)
CK (P)	Sterols (P)	Ceruloplasmin (S)
CRP (P)	Bile acids (U)	Zinc (S, U)
Lactate (P)	Oligosaccharides (U)	Manganese (B)
Glucose (P)	Mucopolysaccharides (U)	Lysosomal Enzymes (S)
Ammonia (B)	VLCFA (P) Polyols (P, U) Immunoglobulins (P) Porphyrins (U, RBC) Lipid panel (S)	Vitamins (S) Flavins (B) SAM & SAH (P) Coagulation factors Glutathione (RBC, P) Dihydroxyacetone phosphate (RBC)

## Data Availability

Data will be made available on request.
