# Supplementary material for: Clinical and biochemical footprints of inherited metabolic disease. XVI. Hematological abnormalities
Source: Mol Genet Metab. Author manuscript; Available in PMC 2025 Jan 22. (PMC11752444; doi:10.1016/j.ymgme.2023.107735)
Supplement: 1 [file NIHMS2044636-supplement-1.docx]

**Supplemental Table S1.** Categories of hematological abnormalities reported in inherited metabolic diseases.

| **Abnormal blood cell morphology** |
| --- |
| Acanthocytosis |
| Achanthocytosis |
| Alder-Reilly bodies |
| Anisocytosis |
| Basophilic stippling |
| Cytoplasmic granules (histiocytes) |
| Granulation in lymphocytes |
| Macrothrombocytes |
| Neutrophils, hypersegmented |
| Pelger Huet bodies |
| Poikilocytosis |
| Sea blue histiocytes |
| Spiculated cells |
| Vacuolated granulocytes |
| Vacuolated granulocytes with lipid droplets (Jordans’ anomaly) |
| Vacuolated lymphocytes |
| **Coagulation abnormalities (bleeding tendency)** |
| Bleeding tendency |
| Coagulation, impaired |
| Coagulopathy |
| Disseminated intravascular coagulation |
| Epistaxis |
| Factor XI |
| Antithrombin III |
| Protein C |
| Perinatal bleeding diathesis |
| Platelet function, abnormal |
| Vit K responsive bleeding |
| **Anemias** |
| Anemia |
| Anemia, dyserythropoietic |
| Anemia, hemolytic |
| Anemia, hypochromic |
| Anemia, hypoplastic, macrocytic |
| Anemia, macrocytic |
| Anemia, megaloblastic |
| Anemia, microcytic |
| Anemia, microcytic, hypochromic |
| Anemia, non-spherocytic, hemolytic with basophilic stippling |
| Anemia, sideroblastic |
| Hemolysis |
| Hemolytic crisis |
| Hemolytic uremic syndrome (atypical) |
| Hypochromia |
| Macrocytosis |
| Microcytosis |
| **Abnormal blood count** |
| Lymphopenia |
| Neutropenia |
| Pancytopenia |
| Thrombocytopenia |
| **Hypercoagulability** |
| Thromboembolic episodes |
| Thromboembolism |
| Thrombosis |
| **Marrow abnormality** |
| Blue histiocytes |
| Bone marrow aplasia |
| Bone marrow hypoplasia |
| Foam cells |
| Hemophagocytosis |
| Iron overload |
| Ringed sideroblasts on bone marrow |
| Sideroblasts (bone marrow) |
| Vacuolization of hematopoietic precusors |
| **Other** |
| Augustine-null blood type |
| Eosinophilia |
| Neutrophilia |
| Methemoglobin (B) |
| Polycythemia |
